# Computational Prediction and Experimental Verification of New MAP Kinase Docking Sites and Substrates Including Gli Transcription Factors

**DOI:** 10.1371/journal.pcbi.1000908

**Published:** 2010-08-26

**Authors:** Thomas C. Whisenant, David T. Ho, Ryan W. Benz, Jeffrey S. Rogers, Robyn M. Kaake, Elizabeth A. Gordon, Lan Huang, Pierre Baldi, Lee Bardwell

**Affiliations:** 1 Department of Developmental and Cell Biology, University of California, Irvine, California, United States of America; 2 Institute for Genomics and Bioinformatics, University of California, Irvine, California, United States of America; 3 Center for Complex Biological Systems, University of California, Irvine, California, United States of America; Max-Planck-Institut für Informatik, Germany

## Abstract

In order to fully understand protein kinase networks, new methods are needed to identify regulators and substrates of kinases, especially for weakly expressed proteins. Here we have developed a hybrid computational search algorithm that combines machine learning and expert knowledge to identify kinase docking sites, and used this algorithm to search the human genome for novel MAP kinase substrates and regulators focused on the JNK family of MAP kinases. Predictions were tested by peptide array followed by rigorous biochemical verification with *in vitro* binding and kinase assays on wild-type and mutant proteins. Using this procedure, we found new ‘D-site’ class docking sites in previously known JNK substrates (hnRNP-K, PPM1J/PP2Czeta), as well as new JNK-interacting proteins (MLL4, NEIL1). Finally, we identified new D-site-dependent MAPK substrates, including the hedgehog-regulated transcription factors Gli1 and Gli3, suggesting that a direct connection between MAP kinase and hedgehog signaling may occur at the level of these key regulators. These results demonstrate that a genome-wide search for MAP kinase docking sites can be used to find new docking sites and substrates.

## Author Summary

Protein kinases are enzymes that regulate key cellular processes by covalently attaching a phosphate group to substrate proteins; they are crucial components of signaling pathways involved in cancer, diabetes, and many other diseases. Identifying the substrates of particular protein kinases is challenging, and many existing biochemical methods are biased against weakly expressed proteins like transcription factors. Here we exploited the observation that mitogen-activated protein kinases (MAPKs) briefly attach to many of their substrates before phosphorylating them, docking onto a sequence known as the ‘D-site’. We developed D-finder, a computational tool that uses a combination of expert knowledge and machine learning to search genome databases for D-sites. We then verified several of D-finder's predictions using rigorous and well-established biochemical assays. The most intriguing predicted and verified substrates were the Gli1 and Gli3 transcription factors of the ‘hedgehog’ signaling pathway. Gli transcription factors are involved in embryonic development and stem cell differentiation, and have also been found to be hyperactive in several types of cancer. There is emerging evidence that crosstalk with MAPK pathways is important in Gli-mediated regulation. Our study, however, is the first to show that MAPKs directly phosphorylate Gli transcription factors.

## Introduction

Protein kinases – enzymes which catalyze covalent addition of phosphate groups to substrate proteins – are essential components of the vast majority of eukaryotic signal transduction and regulatory networks. The human proteome contains just over 500 protein kinases [1], while it has been estimated that at least one-third of all proteins in a typical mammalian cell are phosphorylated [2]. Given these numbers, it is clear that most protein kinases have many different physiological substrates, and that the majority of these substrates remain to be identified.

Many biochemical methods have been developed to identify novel substrates of protein kinases, such as mass-spectrometry, 2D gel electrophoresis, chemical tags used for *in vitro* phosphorylation assays, and others, but most of these methods are biased against weakly expressed proteins (reviewed in [2], [3]). In contrast, computational scanning of genomes to predict novel substrates is blind to protein expression levels, and will also not miss those proteins that are only expressed in rarely studied cell types. The success of such approaches, however, is predicated upon the existence of sufficiently non-degenerate sequence patterns to search for.

Protein kinases phosphorylate serine/threonine or tyrosine residues in proteins, and a few residues on either side of the target phosphoacceptor residue typically also influence kinase-target selection [4], [5]. For example, both cyclin-dependent kinases and mitogen-activated protein kinases recognize a core motif consisting of Ser/Thr-Pro, which is influenced by nearby residues [6]. Phosphorylation-site consensus motifs have been compiled from known examples and from data obtained using peptide libraries [7], [8]. Unfortunately, these motifs are typically short and degenerate, so that they are found in many proteins by chance. Hence, while there have been successes using these motifs to find new substrates (e.g. [9], [10], [11]), this approach has not generally been applied systematically on a genomic level.

Substrate prediction based on target peptide specificity is even more problematic for those kinases that recruit their substrates via interactions outside of the catalytic pocket [12]. Work over the past 15 years or so has established the paradigm that many protein kinases bind with relatively high affinity to interaction motifs on substrates that are distal to the target phosphorylation site(s) [13], [14], [15], and that these interactions can be crucial for efficient signal transmission [16]. This type of “docking” strategy is used extensively in mitogen-activated protein kinase (MAPK) signaling [16], [17]. For example, when the MAP kinase JNK2 phosphorylates its transcription factor target c-Jun, it first tethers itself to a docking site located within residues 30–45 of c-Jun, and then phosphorylates c-Jun on Ser63 and Ser73. Mutation or deletion of this docking site drastically reduces the ability of JNK2 to phosphorylate these residues [18].

**Figure 1:**
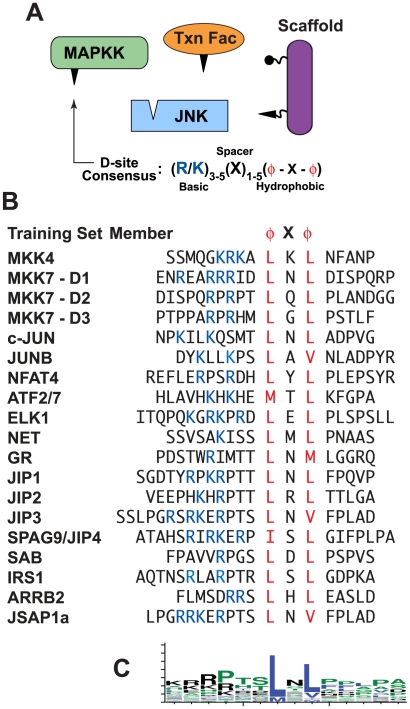
MAP kinases interact with D-sites on substrates and regulators. (A) JNK and several classes of JNK-interacting proteins. The D-site on JNK-binding proteins is shown as a triangle. (B) Literature-verified JNK D-sites that were used as a training set for the hidden Markov model component of D-finder. (C) Sequence logo [111] of the central 14 residues of the D-sites shown in B.

Although there are several classes of MAPK-docking site, many of the known substrates of MAP kinases contain a docking motif known as the ‘D-site’ (see Fig. 1). D-sites are also found in MAPK-regulating proteins such as MAPK kinases (MKKs), scaffold proteins and MAPK phosphatases. The D-site consensus consists of a basic cluster of 1–4 residues, a short spacer, and a hydrophobic-X-hydrophobic submotif. Crystallographic and mutagenesis studies have established that D-sites on substrates and regulators bind in an extended fashion to complementary surface patches and grooves on their cognate MAPKs [19].

MAPKs are essential components of eukaryotic signal transduction networks that enable cells to respond appropriately to growth factors, differentiation cues, stresses, and other signals [20]. MAPK pathways are dysregulated in many diseases, including cancer, developmental disorders, degenerative diseases (e.g. Alzheimer's, Parkinson's, Huntington's, muscular dystrophy) and metabolic disorders (e.g. obesity and diabetes) [21], [22]. Many substrates of MAPKs have been identified [23], [24], but given the widespread involvement of MAPKs in fundamental and disease processes, it is suspected that many more remain to be found [25]. In addition, MAPK target phosphosites are too degenerate to use for genome-scale screening [26]. With this in mind, we developed a program to search genome sequences for putative D-sites, with the aim of identifying novel MAPK substrates and regulators. Here we report the success of this approach, when applied to the human genome, both in identifying previously unknown D-sites in known substrates, and in discovering novel D-site-containing MAPK substrates.

## Results

### An algorithm for detecting MAPK-docking sites

In mammalian cells, four major MAPK pathways have been characterized: ERK1/2, JNK, p38, and ERK5 [27], [28]. D-site-mediated interactions are used extensively by the first three of these [29], and perhaps by ERK5 as well [30]. While some D-sites show selectivity in their ability to bind to either the ERK, JNK or p38 families of MAPKs [31], other D-sites bind to members of more than one MAPK family [17]. This suggests that it is both possible and desirable to devise a search procedure that can utilize family-specific information, and also suggests that there is hidden information, outside the core consensus, that dictates family preferences. For these reasons we decided to focus on developing a search procedure that could identify novel substrates and regulators of the JNK family of MAPKs.

We believed that our recent characterization of four new JNK D-sites [32], [33] had pushed the number of literature-verified sites towards the critical mass needed to implement a machine-learning approach [34]. A profile Hidden Markov Model (HMM) architecture, which statistically represents a pattern of position-specific conservation for a series of related sequences, is a probabilistic machine learning approach that has the potential to discover patterns in sets of data that are difficult to notice by direct observation [35]. HMMs have proven useful, for example, in sequence alignment of protein families and prediction of novel family members [36], [37], prediction of signal peptides [38], and prediction of p53-binding sites [39]. HMMs also form the foundation of Pfam's homology searching capabilities [40]. Within the profile HMM architecture, a “training set” of validated sequences is used to update a set of emission and transition probability matrices. Following enough iterations to converge each parameter's value, the HMM can be used to compute the probability that any test sequence is related to the training set. Essentially, the computer infers what the sequences in the training set have in common, and then evaluates the probability that a test sequence will be generated by the same rules it has inferred from the training set [41], [42], [43].

To evaluate the utility of an HMM-based approach for finding novel D-sites, a list of 20 proteins containing functionally verified JNK-docking sites was compiled from our own results and the work of many others [17], [18], [44], [45], [46], [47], [48], [49], [50], [51], [52], [53], [54], [55], [56] (Fig. 1B). Members of the training set included D-sites from the JNK activating kinases MKK4 and MKK7 [32], [33], JNK scaffold proteins such as JIP1-3, JIP4/SPAG9, JSAP1, and beta-arrestin2 [46], [47], [48], [49], [55], [56], and JNK substrates such as the transcription factors c-Jun, ATF2, Elk-1 and Net [18], [45], [54]. We limited the training set to experimentally-validated JNK-docking sites, and excluded those D-sites known to bind preferentially to ERK or p38 over JNK, as well as those which had not been tested with JNK. We also excluded MAPK phosphatases from our training set because of evidence that they use an extended docking motif [57]. This training set was used to train a profile HMM architecture designated ‘D-learner’ (see Methods for further details); the trained HMM was designated D-learner.T1 (short for ‘D-learner trained with Training Set #1’).

### Validation of the HMM algorithm

A training set of only 20 members is close to the minimum number needed to derive a useful HMM; this small set was necessitated by our decision to limit the training set only to those D-sites that were literature verified. Therefore, several tests were carried out to assess the ability of D-learner.T1 to accurately discriminate D-sites from other sequences.

First, the probabilistic “Viterbi score” (see Methods) given by D-learner.T1 to each member of the training set was computed. The HMM gave the best score to training set members JIP1/MAPK8IP1 and JIP2/MAPK8IP2 (2.4E-14 and 3.3E-17, respectively), and the ‘worst’ score to members IRS1 and GR (5.0E-26 and 1.5E-26, respectively). In contrast, randomly-permuted sequences derived from training set members generated scores ranging from 1E-24 to 1E-36. Note that the closer a Viterbi score is to 1, the ‘better’ it is (where better scoring sequences presumably have a higher probability of being a *bona fide* D-site), yet very good scores may still be very small numbers; this is standard in HMM-based approaches [42].

**Figure 2:**
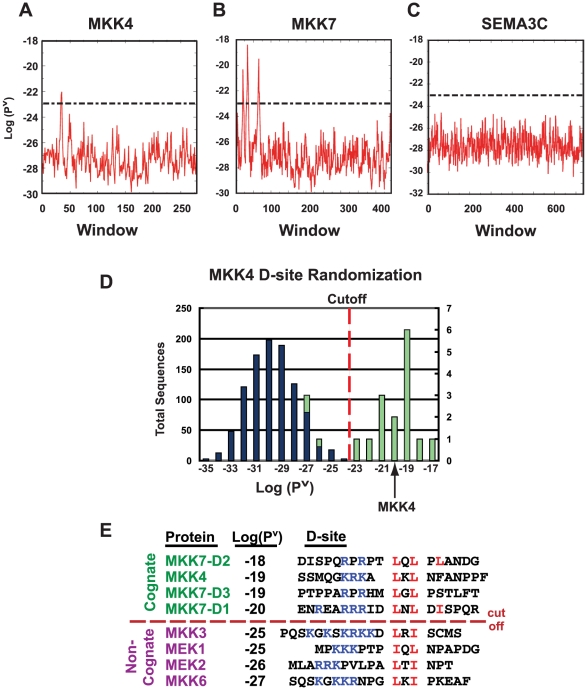
Validation of the D-learner hidden Markov model. (A–C). The HMM accurately identifies known D-sites in full-length sequences. Full-length sequences run through the HMM give a Viterbi probability for every window tested. The x-axis displays the window number and the y-axis shows the log of the Viterbi probability for each window. The dashed lines represent the threshold of E-23 for a window to be considered a predicted D-site. MKK4 (A) has one peak, MKK7 (B) has three peaks, and the arbitrarily-chosen full length sequence SEMA3C (C) has zero peaks above the threshold. (D) The HMM does not score randomized sequences highly, even if they have the same composition as a high-scoring D-site. Histogram of scores assigned to 1,000 scrambled sequences with same sequence composition as the MKK4 D-site (blue, left ordinate labels) and the 20 training set D-site sequences (green, right ordinate labels). Sequences were binned by score, with no sequences scoring below −37 or above −14. For the MKK4 randomized set, zero sequences surpassed the −23 threshold (dashed line). For the 20,000 total randomized D-site sequences, 30 sequences (0.15%) scored above this threshold. For the training set, 16 sequences (80%) surpassed the E-23 threshold. (E) The HMM scores JNK D-sites higher than D-sites selective for ERK- or p38-family MAPKs. The name, D-learner-assigned score, and sequence of all known human MKK D-sites are shown. The JNK D-sites (MKK4 and the 3 MKK7 D-sites) surpass the −23 threshold; however, the non-cognate D-sites, although they contain the core consensus basic (blue) and hydrophobic (red) residues, do not score above the threshold.

Next, the full-length sequences of the proteins in the training set were tested. In each case, the HMM was able to identify the published D-site as the top scoring window within the full-length polypeptide. Graphs of the scores for successive windows running from the amino- to the carboxy- terminus in three test proteins are shown in Fig. 2. The single D-site in MKK4 [32] resulted in a single peak near the N-terminus of this protein (Fig. 2A). Moreover, the three known D-sites in the N-terminus of MKK7 [33] resulted in three corresponding peaks in the D-learner.T1 output (Fig. 2B). In contrast, an arbitrarily chosen coding sequence with no known D-sites has no obvious peaks (Fig. 2C). This arbitrarily chosen sequence is representative of any of over 30,000 sequences in the translated human transcriptome in which D-learner.T1 did not find any window with a score above 1E-23 (this cutoff is represented by the dashed lines Fig. 2A–D).

Third, as a test of the ability of D-learner to identify literature-verified D-sites that it had not been trained on, a leave-one-out cross-validation (LOOCV) test was implemented. In this test, each of the original 20 training set sequences was removed from the training set one at a time (so that 19 D-sites remained in the training set), the HMM was retrained, and the removed sequence (in the context of its full length protein sequence) was used as a test sequence. In every case, the removed sequence was still the top scoring window within the full-length polypeptide (data not shown). It should be noted that the LOOCV test is most stringent when the left out sequence is not overly similar to the training sequences, as was true in many but not all of the LOOCV tests we performed (see Text S1 Fig. 1 for a multiple sequence alignment of the training set D-sites). We also performed a combinatorial series of “leave-four-out” cross validation tests. In these tests the ability of D-finder to identify the *bona fide* D-sites in the left-out sequences degraded to 70%. This is evidence that the 20-member training set is indeed near the minimum required for an effective HMM.

As a fourth test, we assessed the range and distribution of probabilities that D-learner.T1 assigned to scrambled D-site sequences. Each of the D-sites in the training set was randomly permuted 1,000 times and scored by the HMM. Fig. 2D shows the result obtained when the randomized strings of MKK4 were binned and plotted. The median of the 20,000 total sequences was 2.5E-30, with a minimum score of 6.2E-37 and a maximum of 2.2E-19. This can be compared to the scores given to the unrandomized training set of 20 sequences, which had a median, minimum and maximum of 6.2E-20, 1.5E-26 and 2.4E-14, respectively. Thus, D-learner.T1, on average, assigned much better scores to bona fide D-sites than to scrambled sequences of the same composition.

Next, we used receiver operating characteristic (ROC) analysis, which compares how the true positive and false positive rates vary as the threshold used to discriminate predicted positives from predicted negatives is varied. As a set of true positives, we used the training set members. To estimate the false positive rate as a function of threshold, we ran D-learner.T1 on the predicted proteomes of the bacteria *E. coli* and *B. subtilis*. As bacteria do not contain MAP kinases, any D-site-like sequences found in these organisms either occur by chance or have evolved to serve some other function. In either case, they can be considered false positives. The results of this analysis are shown in Text S1 Fig. 2. The area under the curve is 0.92, where 1.0 would be the score of a perfect classifier, and 0.5 would be the score obtained by flipping a coin to classify each window. A score of 0.92 thus indicates very good performance.

Based on these data, we set the threshold for a top scoring subsequence to be considered a “high-quality” predicted D-site to 1E-23. This threshold represents a compromise between the goals of (1) including members of the training set while (2) excluding all but the very tail of the scrambled distribution. The threshold of 1E-23 is a point that only 0.15% (30/20,000) of the scrambled sequences surpassed, yet 80% (16/20) of the training set members did. In addition, less than 0.005% (1/30,000) of 19mers randomly chosen from either the human, *E. coli* or *B. subtilis* proteomes surpassed this threshold. This threshold is represented by the dashed lines in the graphs in Fig. 2A–D.

As a final assessment of D-learner.T1, we compared the scores it gave to cognate vs. non-cognate D-sites. MKK4 and MKK7 are the physiological (cognate) activators of JNK1-3, whereas the MAPK kinases MEK1/2 and MKK3/6 activate the ERK1/2 and p38 MAP kinases, respectively, and do not phosphorylate JNK1-3. Consistent with these strong enzymatic preferences, JNK proteins bind selectively to MKK4- and MKK7-derived D-sites and do not bind appreciably to D-sites in other MKKs [31], despite the observation that all MKK-derived D-sites share the core consensus basic and φ-X-φ motifs (Fig. 2E). In accord with these biochemical results, D-learner.T1 ranked the D-sites in the cognate JNK kinases MKK4 and MKK7 much higher than those in non-cognate MAPKs. The cognate MKK4 D-site and all three cognate MKK7 D-sites had Viterbi scores ranging between 1E-18 to 1E-20, whereas the non-cognate D-sites in MEK1/2 and MKK3/6 had lower scores ranging between 1E-25 and 1E-27. In addition, all four cognate D-sites scored well above the cut-off of 1E-23, whereas all four non-cognate D-sites scored below this cut off. Thus, the trained hidden Markov model was able to discriminate JNK-docking sites from docking sites for other MAP kinases.

### Development of a hybrid algorithm

**Figure 3:**
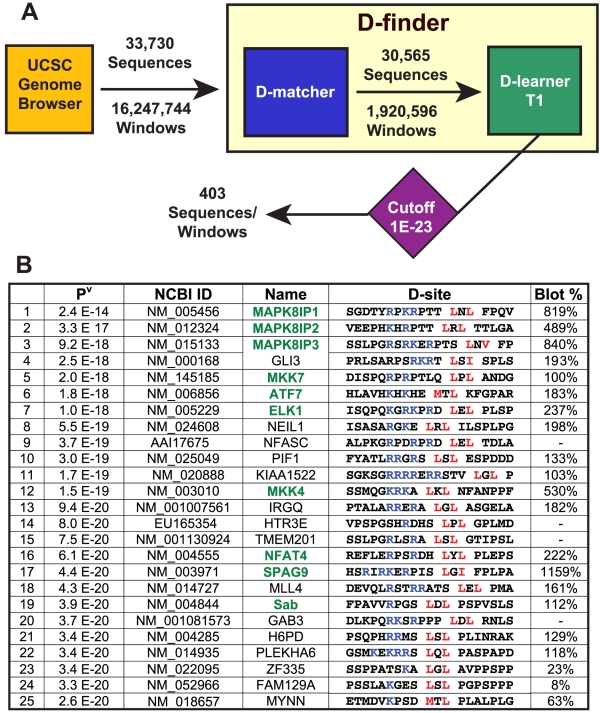
D-finder architecture and results of human genome search. (A) Overview of D-finder. D-finder consists of D-matcher, a pattern matching algorithm employing expert knowledge, and D-learner.T1, a profile HMM trained on the training set shown in Fig. 1B. D-matcher filters out most windows, but found many acceptable windows in most sequences. D-learner assigns a probability score to each window it is passed, and found above-threshold windows in only 403 of the sequences passed to it by D-matcher. When D-learner was run without D-matcher interceding, it found 2,260 above-threshold windows in 1,784 sequences. (B) The top 25 D-sites found by D-finder in the human genome.

To explore the potential utility of D-learner.T1 to predict novel D-sites from genome-scale information, it was run on the translated human transcriptome. Examination of the top-ranked sequences revealed a potential weakness in the D-learner.T1 model: it was giving high-ranking scores to many sequences that did not contain a canonical φ-X-φ submotif, and in some cases also to sequences lacking even a single-residue basic submotif (see Text S1 Fig. 3). Mutagenesis studies have shown that the basic submotif, as well as both hydrophobic residues in the φ-X-φ submotif, are crucial for D-site function [32]. Therefore, we concluded that sequences that did not contain a canonical basic or φ-X-φ submotif would be enriched for false positives relative to those that did.

In parallel with the development of D-learner, we also developed an expert knowledge-based pattern-matching algorithm that we dubbed D-matcher. The first version of D-matcher searched for φ-X-φ submotifs and appropriately-spaced clusters of basic residues (see Methods). D-matcher performed well in several validation tests, but produced too many sequences with similar scores. Also, we believed that there was additional information contained within D-site sequences that was difficult to incorporate into a rule-based algorithm. It was therefore deemed unsuitable for genome-scale screening on its own.

Because neither D-learner nor D-matcher was ideal for genome-scale screening as separate algorithms, we developed a hybrid program named D-finder, which incorporates a simplified D-matcher as a pre-screen for sequences suitable to pass to D-learner. This program was used for all subsequent analyses.

### Transcriptome screening

To identify putative novel JNK-interacting proteins in the human genome, 33,730 full-length protein sequences, representing the predicted human translated transcriptome, were scored by D-finder (Fig. 3A). Overall, 403 proteins contained predicted D-sites that surpassed our conservative threshold (see Table S1 Table 1), corresponding to a hit rate of 1.19%. To provide a basis for comparison, we also ran D-finder on the predicted proteomes of the bacteria *E. coli* and *B. subtilis*, obtaining per protein hit rates of 0.58% and 0.36%, respectively.

The top 25 predicted human D-sites sequences, ranked according to Viterbi score, are shown in Fig. 3B. Six of the top seven and ten of the top nineteen are training set members (colored green in Fig. 3B), indicating that the literature-verified D-sites were given an appropriately high ranking in the context of a whole genome search. In general, predicted human D-sites sequences were conserved in the orthologous mouse sequences (median D-site sequence identity 89%, see Table S1 Table 2), in those cases where orthologs could be readily discerned.

Scansite is a published motif-finding tool that uses a weight matrix-based scoring algorithm to search for many sequence motifs, including ERK D-sites [7]. Since ERK D-sites share a core consensus with JNK D-sites, we compared Scansite to D-finder. Comparing the highest scoring D-site when each tool was run on the same sequence revealed that D-finder and Scansite often prioritized the same site. For example, for the proteins in the D-finder training set, Scansite found the same D-site as D-finder in 65% of the cases. When we compared their performances in database searches, however, Scansite and D-finder were quite different. For example, only one of the top 25 predictions from D-finder was also among the top 25 predictions of Scansite, and only three of D-finder's top 25 predictions were in Scansite's top 500 predictions. Notably, none of the four proteins analyzed in detail below (hnRNP-K, PPM1J, Gli3 and Gli1) were in Scansite's top 2000. Thus, Scansite and D-finder prioritize different sequences.

### Identification of a D-site in hnRNP-K

The list of predictions generated by D-finder was first scanned for known JNK substrates or regulators that were not previously known to contain a D-site. Heterogeneous nuclear ribonucleoprotein K (hnRNP-K) is an RNA and DNA binding protein that regulates transcription and translation [58], [59], and has been implicated in the pathology of several types of cancer [60], [61]. It is also a known substrate of several different kinases, including both JNK and ERK. Although ERK and JNK phosphorylation have been shown to modulate the regulatory activities of hnRNP-K [61], [62], [63], and key MAPK phosphorylation sites on hnRNP-K have been mapped [62], [63], no MAPK-docking sites have heretofore been identified on hnRNP-K. D-finder assigned a Viterbi score of 4.5E-22 to a putative D-site (core sequence: RGGSRARNLPL) that it found at residues 296–310 of hnRNP-K; overall, this D-site ranked 206 of the 403 above-threshold sites.

**Figure 4:**
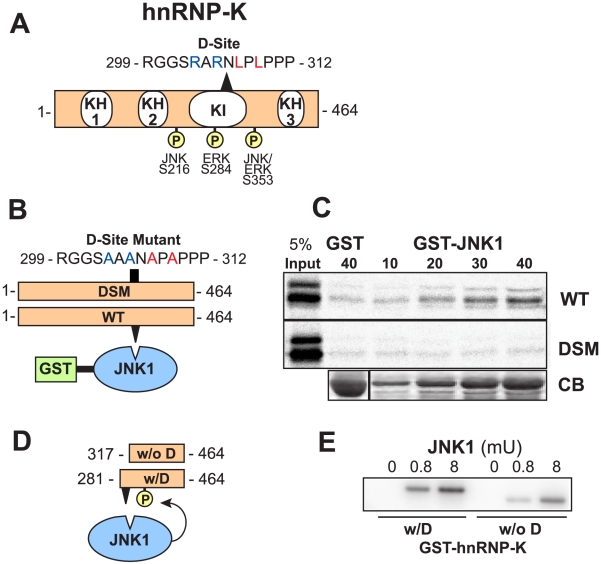
Identification of a D-site in the known JNK substrate hnRNP-K. (A) Full-length hnRNP-K protein. KH, K homology domain; KI, K interaction domain. The positions of known JNK and ERK phosphosites and the D-finder-predicted D-site are shown, with key consensus basic (blue) and hydrophobic (red) residues highlighted by color. (B) Wild-type (WT) and D-site mutant versions (DSM) of hnRNP-K were tested for binding to GST-JNK1. The sequence of the D-site mutant is shown. (C) As shown in B, ^35^S-radiolabeled full-length hnRNP-K protein and a D-site mutant derivative were prepared by *in vitro* translation and partially purified by ammonium sulfate precipitation, and portions (5% of the amount added in the binding reactions) were resolved on a 10% SDS-polyacrylamide gel (lane 1). Samples (∼1 pmol) of the same proteins were incubated with 40 µg of GST (lane 2) or with 10 to 40 µg of GST-JNK1 (lanes 3–6), bound to glutathione-Sepharose beads, and the resulting bead-bound protein complexes were isolated by sedimentation and resolved by 10% SDS-PAGE on the same gel. The gel was analyzed by staining with Coomassie Blue (CB) for visualization of the bound GST fusion protein (a representative example is shown in the lowest panel) and by Phosphorimager analysis for visualization of the bound radiolabeled protein (upper two panels). (D) Fragments of hnRNP-K were tested as substrates for *in vitro* phosphorylation by active JNK. (E) As shown in D, GST fusions to hnRNP-K_281–464_ (containing the D-site, w/D) and hnRNP-K_317–464_ (deleted of the D-site, w/o D) were purified and incubated with purified active JNK1 and [γ-^32^]ATP for 20 min. Substrate concentration: 500 nM; Enzyme activity: 0, 0.8 mU, or 8mU. Reaction products were separated by SDS-PAGE and incorporation of radioactive phosphate into the substrate was assessed on a PhosphorImager.

A diagram of hnRNP-K with its three RNA/DNA-binding K homology (KH) domains is shown in Fig. 4A [58]. The predicted D-site lies within the K-protein-interactive (KI) domain, a region known to mediate protein-protein interactions with several other hnRNP-K binding partners [58], [64], [65], [66].

To assess the nature of the interaction between human JNK1 and human hnRNP-K, JNK1 was fused at its N terminus to *Schistosoma japonicum* glutathione *S*-transferase (GST), and the resulting fusion protein was expressed in bacteria and purified by adsorption to glutathione-Sepharose beads. JNK1 prepared in this manner is obtained in its unphosphorylated, unactivated state. GST-JNK1 (or GST alone as a negative control) was then incubated with full-length human hnRNP-K that had been produced in radiolabeled form by *in vitro* translation (Fig. 4B). Bead-bound complexes were collected by sedimentation, washed extensively, and analyzed by SDS-PAGE and autoradiography. As shown in Fig. 4C, full-length hnRNP-K bound efficiently to JNK1; this binding was specific, because only trace precipitation of hnRNP-K occurred when GST was used instead of the GST-JNK1 fusion protein.

To test the hypothesis that the predicted D-site in hnRNP-K is important for JNK binding, the ability of wild-type full-length hnRNP-K to bind to JNK1 was compared to a D-site mutant of hnRNP-K, in which the critical basic and hydrophobic residues of the D-site consensus were mutated to alanine (Fig. 4B). As shown in Fig. 4C, the D-site mutant of hnRNP-K did not bind to JNK1 above background. Thus, the predicted D-site in hnRNP-K mediates binding to JNK1.

The predicted D-site lies within 100 residues of mapped JNK and ERK phosphorylation sites in hnRNP-K (Fig. 4A) [57], [58]. Having established that this D-site mediated JNK binding to hnRNP-K, we next wanted to determine if it also facilitated the phosphorylation of hnRNP-K by JNK at the known JNK target phosphosite Ser353. To limit the possible phosphoacceptor sites to Ser353, two N-terminally truncated versions of hnRNP-K, one with the D-site (w/D, residues 281–464) and one without (w/o D, residues 317–464), were fused to GST and expressed and purified from bacteria. These two protein substrates were incubated with purified active JNK1 and radiolabeled ATP in a standard *in vitro* kinase assay (Fig. 4D). Active JNK1 efficiently phosphorylated the GST-hnRNP-K fragment containing an intact D-site (Fig. 4E). In contrast, the level of phosphorylation was significantly diminished (approx 9-fold) for the GST-hnRNP-K fragment lacking the D-site. GST alone was not phosphorylated by any of the active MAPK enzymes used in this work (data not shown).

Because hnRNP-K is also a substrate of ERK2, we tested the ability of ERK2 to bind to and phosphorylate hnRNP-K in a D-site-dependent manner. GST-ERK2 bound to hnRNP-K, and the strength this interaction was reduced about 3-fold by mutation of the D-site (data not shown). In addition, active ERK2 phosphorylated the GST-hnRNP-K fragment, and the extent of phosphorylation was slightly reduced by removal of the D-site (data not shown). The simplest explanation of these results is that ERK2 utilizes the predicted D-site, but that there is also an additional docking site for ERK somewhere within hnRNP-K residues 281–464. This would not be surprising, as many MAPK substrates and regulators have been shown to contain multiple MAPK-docking sites [33], [54], [67]. A second possibility is that ERK2 phosphorylation of Ser353 is largely independent of ERK2-hnRNP-K docking. We did not examine whether the D-site promoted ERK-mediated phosphorylation of Ser284 or JNK-mediated phosphorylation of Ser216.

To summarize, D-finder predicted a novel D-site in the known JNK substrate hnRNP-K; this D-site was found to be a *bona fide* D-site that mediated JNK-hnRNP-K binding and promoted JNK-dependent phosphorylation of Ser353 in hnRNP-K.

### Identification of a D-site in PPM1J/PP2Czeta

PP2Czeta, also known as PPM1J, is type 2C protein phosphatase that is enriched in testicular germ cells [68]. PPM1J is a recently identified JNK substrate; JNK phosphorylates PPM1J on Ser92 and Thr205, and more weakly on Thr202. Moreover, JNK phosphorylation of Ser 92 of PPM1J reduces its phosphatase activity [69]. No MAPK-docking sites have been reported in PPM1J. D-finder assigned a Viterbi score of 2.7E-22 to a putative D-site (core sequence: RPTFLQL) that it found in residues 68–74; overall, this D-site was rank 256 of the 403 above-threshold sites.

**Figure 5:**
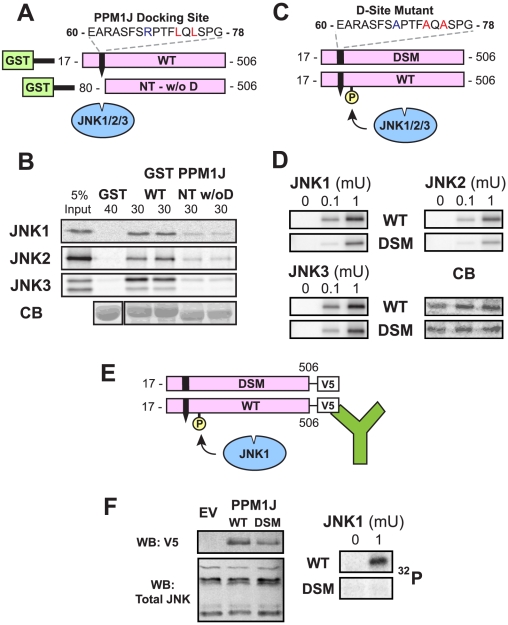
Identification of a D-site in the PPM1J phosphatase. (A) Wild-type (WT) and N-terminal truncated versions (NT w/o D) of PPM1J were fused to GST and tested for binding to JNK 1–3. The sequence of the D-site is shown. (B) As shown in A, ^35^S-radiolabeled JNK1, 2 or 3 (∼1 pmole) were tested for binding to 40 µg of GST (lane 1) or 30 µg of GST-PPMIJ K_17–506_ (containing the D-site, lanes 3 and 4) or GST-PPMIJ K_80–506_ (lacking the D-site, lanes 5 and 6). Lane 1 shows a 5% of the total JNK input. The lower panel shows Coomassie Blue (CB) staining of the sedimented GST-fusion proteins. Other details as in Fig. 4C. (C) Wild-type (WT) and D-site-mutant (DSM) versions of PPM1J were tested as substrates for *in vitro* phosphorylation by active JNK1-3. (D) As shown in C, 1 µM of each GST-PPM1J protein was incubated with 0, 0.1 or 1 mU JNK1-3 and [γ-^32^]ATP for 20 min. Incorporation of radioactive phosphate into the substrate, as assessed by autoradiography, is shown in 3 panels, and a representative Coomassie blue (CB) stained gel, to demonstrate equal loading of the substrates proteins, is shown. (E) Wild-type and D-site mutant versions of PPM1J were C-terminally-tagged with the V5 epitope, expressed in Cos-1 cells, immunoprecipitated, and used as substrates for JNK1-mediated phosphorylation. (F). As shown in E, Cos-1 cells were transfected with either empty vecor (EV), PPM1J-V5 WT, or PPM1J-V5 DSM. 16 h post-transfection, the cells were harvested, lysed, and immunoprecipitated with anti-V5 antibodies. MAPK buffer, [γ-^32^]ATP, and active JNK1 were added to the immunoprecipitated pellets, and phosphorylation of the immunoprecipitated proteins was visualized by PhosphorImager. In addition, portions (20 µg) of each lysate were separated by SDS-PAGE and immunoblotted (WB, for Western Blot) with either anti-V5 (1∶5000) or anti-total JNK (1∶500) antibodies.

To test the ability of this D-site to mediate the binding of PPM1J to JNK *in vitro*, JNK binding to wild-type GST-PPM1J was compared with binding to a PPM1J N-terminal deletion mutant lacking the putative D-site (Fig. 5A). As shown in Fig. 5B, radiolabeled JNK1, JNK2 and JNK3 all bound to wild-type PPM1J but not to GST alone. In comparison, the binding of all three JNK proteins to the PPM1J derivative lacking the D-site was substantially reduced.

To determine if the novel D-site promoted the phosphorylation of PPM1J by JNK, the GST-fused wild-type PPM1J protein and a GST-fused D-site point mutant derivative (DSM) were incubated with active JNK1, JNK2, and JNK3 and radioactive ATP in a standard kinase assay (Fig. 5C). The results of these assays (Fig. 5D) show a significant reduction in phosphorylation of the D-site mutant protein relative to the wild type, at two different concentrations for each of the three JNK enzymes.

As an alternative means to assess D-site-mediated phosphorylation of PPM1J by JNK, V5-epitope-tagged versions of the wild-type and D-site point mutant of PPM1J were expressed in Cos-1 cells and immunoprecipitated with an anti-V5 antibody. Immunoprecipitates were then mixed with purified active JNK1 and radioactive ATP (Fig. 5E). As shown in Fig. 5F, under these conditions wild-type PPM1J was phosphorylated while the D-site-mutant PPM1J was not.

To summarize, D-finder predicted a novel D-site in the known JNK substrate PPM1J; this was found to be a *bona fide* D-site promoting JNK binding and JNK-mediated phosphorylation.

### Verification of novel candidates by peptide array

**Figure 6:**
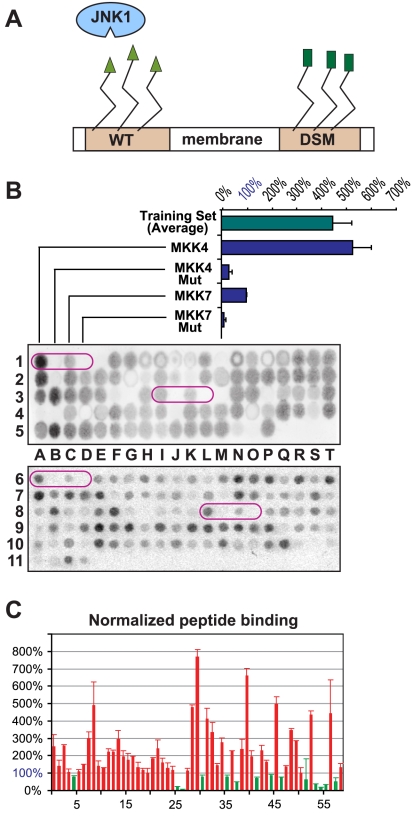
Testing predicted D-sites with peptide arrays. (A) Membrane-attached peptides were probed for binding to radioactively-labeled JNK1. Peptides containing functional JNK-docking sites (e.g. the MKK4 or MKK7-D2 positive controls or accurate predictions) bound to JNK1, while those containing non-binding peptides (e.g. negative controls or false predicitions) did not. (B) Representative examples of peptide arrays probed with ^35^S-labeled JNK1 and then visualized and quantified by Phosphorimager. Controls (circled, in duplicate on each membrane) are the published D-sites of MKK4 and MKK7-D2, and their mutants with alanine substitutions at the critical basic and hydrophobic residues. The binding efficiency of the average of the training set peptides and the positive and negative controls are plotted; this has been normalized by setting the efficiency of the MKK7-D2 positive control to 100%. (C) Plot of the normalized binding percentages (with S.E.M. bars) for the 59 predicted D-site peptides that were tested. The threshold for classification as positive is 100%. Red-colored bars are above threshold, green-colored bars are below threshold.

To begin to weed through the novel D-finder predictions resulting from the transcriptome search, a peptide array approach was employed. Peptide arrays are macro arrays of short peptides that are tethered to a nitrocellulose membrane via a chemical linker attached to their C-termini [70]. Peptides (17-mers) representing the training set members, as well as 59 of the predicted novel D-sites, were arrayed in duplicate and probed for binding to radiolabeled JNK1 (Fig. 6A, 6B, see also Table 3 in Table S1). Each array contained two positive control peptides (MKK4 and MKK7-D2, where MKK7-D2 is the second of the three D-sites in the N-terminal domain of MKK7 [33]) and two negative control peptides (D-site mutants of MKK4 and MKK7-D2, in which the critical basic and hydrophobic residues of the D-site consensus were changed to alanine) (Fig. 6A). Binding of radiolabeled JNK1 to the arrayed peptides was quantified with a PhosphorImager and normalized to the signal obtained with the MKK7-D2 positive control. MKK7-D2 was chosen as a normalization base because it is known to exhibit real, but relatively weak, binding [33], and gave a correspondingly weak signal in the peptide array assay (Fig. 6B). Thus, we judged that any D-site peptide giving a lower binding signal than MKK7-D2 was probably not worth pursuing.

PhosphorImager images of two peptide arrays are shown in Fig. 6B. The results of these experiments highlight the utility of this approach. First, duplicate spots on the array exhibited similar levels JNK1 binding (e.g., see control peptides, circled). Moreover, comparable results were obtained when the same peptide sequences were probed on different arrays on different days (compare control peptides between top and bottom arrays). The MKK4 and MKK7-D2 peptides both exhibited reproducible positive signals on the arrays, with the MKK4 peptide binding more efficiently, consistent with its lower *K_d_* [33]. As anticipated, the D-site mutant negative control peptides exhibited barely detectable binding. Finally, the other members of the training set bound almost as efficiently, on average, as the MKK4 D-site. Thus, the peptide array methodology appeared to provide reproducible and quantitative data on the binding of MAPKs to D-site peptides. Furthermore, signal strength roughly correlated with binding affinities measured by other methods. These conclusions are consistent with our previous experiences using this approach [32], [71], [72].

Fig. 6C shows the normalized JNK binding of 59 high scoring D-finder-predicted D-sites (see Fig. 3B for scores of top 25 predictions and Table 3 in Table S1 for further annotation). These peptides correspond to novel D-finder predictions and are not members of the training set. Of these 59 peptides, 45 bound to JNK1 at a level that was greater than MKK7-D2, our cutoff for weak-yet-real binding. Thus, the predictive accuracy of D-finder was ∼76% (45/59). From these results it can be concluded that the D-finder algorithm is effective at discovering novel peptides that have the ability to bind to JNK.

### Gli3 is a MAPK substrate

Selected candidates that gave a positive binding signal on the peptide array were chosen for further analysis. We first focused on the zinc-finger transcription factor GLI-Kruppel Family Member 3 (GLI3), which contained a D-site that was ranked 4th overall by D-finder, the highest scoring prediction that was not a member of the training set (see Fig. 3B). This D-site (core sequence: RKRTLSI) comprises residues 290–296 of the 1580 residue Gli3 protein. A peptide version of this D-site bound to JNK1 at 193% the level of the MKK7-D2 positive control in the array assay. GLI3 encodes a transcription factor in the hedgehog signaling pathway, and is homologous to the *Drosophila* gene/protein *cubitus interruptus* (Ci) [73], [74]. Germ line mutations in GLI3 have been implicated in two human developmental disorders: Pallister-Hall syndrome and Grieg cephalopolysyndactyly syndrome [75]. Furthermore, several recent studies strongly suggest the possibility of crosstalk between MAPK signaling and hedgehog signaling, but the molecular basis for this crosstalk remains to be identified [76], [77], [78], [79], [80], [81], [82]. For all these reasons, Gli3 protein was chosen for further analysis.

**Figure 7:**
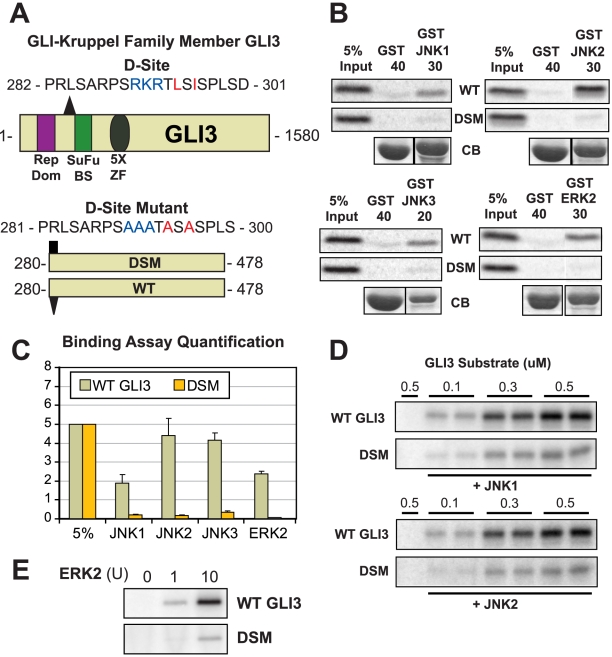
Gli3 is a novel MAP kinase substrate with a functional D-site. (A) Diagram of full length Gli3, shown with its transcriptional repressor domain (Rep Dom, purple rectangle), the Suppressor-of-Fused binding site (SuFu BS, green rectangle), and its 5 Zinc Finger (ZF) DNA-binding domains (gray oval). The position (triangle) and sequence of the D-finder-predicted D-site is also shown. Below, the Gli3_280–478_ wild-type (WT) and D-site mutant (DSM) fragments used for binding and kinase assays are shown, along with the sequence of the D-site mutant. (B) The Gli3_280–478_ wild-type and D-site mutant proteins were tested for binding to GST, GST-JNK1, GST-JNK2, GST-JNK3, and GST-ERK2 (40, 30, 30, 20 and 30 µg respectively). The upper two panels show the bound Gli3 derivatives, with 5% if the total input shown in lane 1; the lower panel shows Coomassie Blue (CB) staining of the sedimented GST-fusion proteins. Other details as in Fig. 4C. (C) Graph of the results of three independent repetitions of the binding assay shown in A and B, with duplicate points in each repetition. *Standard error* bars are shown (n = 3). (D) *In vitro* kinase assays assessing the phosphorylation of the WT and DSM fragments of Gli3 by active JNK1 and JNK2. Three separate concentrations of substrate (0.1, 0.3 and 0.5 µM) were incubated with 0.5 mU (∼1 ng) of active enzyme. Image is representative of three independent experiments. Other details as in Fig. 4E. (E) *In vitro* kinase assay assessing the phosphorylation of the WT and DSM fragments of Gli3 by activated ERK2. Substrate concentration: 0.5 µM. Enzyme activity: ERK2 – 0, 1, or 10 units (10 units is ∼1 ng).

As large proteins often exhibit non-specific binding in pull-down assays, we constructed a fragment consisting of amino acids 280–478, stretching from the D-site to just before the beginning of the five kruppel zinc-finger domains (Fig. 7A, bottom panel). At the same time, we constructed a D-site mutant version of this protein (DSM), changing the key basic and hydrophobic residues in the D-site to alanine (Fig. 7A). Both versions were produced by *in vitro* transcription/translation and assessed for binding to GST-fused JNK1, JNK2 and JNK3. As shown in Fig. 7B and 7C, the wild-type protein fragment bound well to all three JNK paralogs, with a slight preference for JNK2 over JNK1/3. In contrast, the D-site mutant exhibited greatly decreased binding to all three JNK proteins. Thus the D-site in Gli3 binds to JNKs as predicted.

Although D-finder was trained with JNK docking sites and showed an ability to discriminate JNK D-sites from ERK and p38 D-sites (see Fig. 2E), several of the D-sites in the training set are known to bind to ERK as well as JNK, e.g. the Elk-1 D-site [45]. Therefore, wild-type and D-site mutant Gli3 were tested for binding to ERK2. Indeed, Gli3 also bound ERK, while the D-site mutant did not (Fig. 7B, 7C).

We hypothesized that the newly identified D-site might promote the MAPK-mediated phosphorylation of Gli3. To test this possibility, GST-Gli3_280–478_ WT and DSM proteins were purified and used as substrates for *in vitro* kinase assays with purified activated MAPKs. As shown in Fig. 7D and 7E, wild-type Gli3 was an efficient substrate for all 3 MAPKs tested (JNK1, JNK2 and ERK2), whereas the D-site mutant exhibited greatly reduced phosphorylation. Thus the D-site promotes MAPK-mediated phosphorylation of Gli3.

### Identification of a target phosphosite for JNK on Gli3

**Figure 8:**
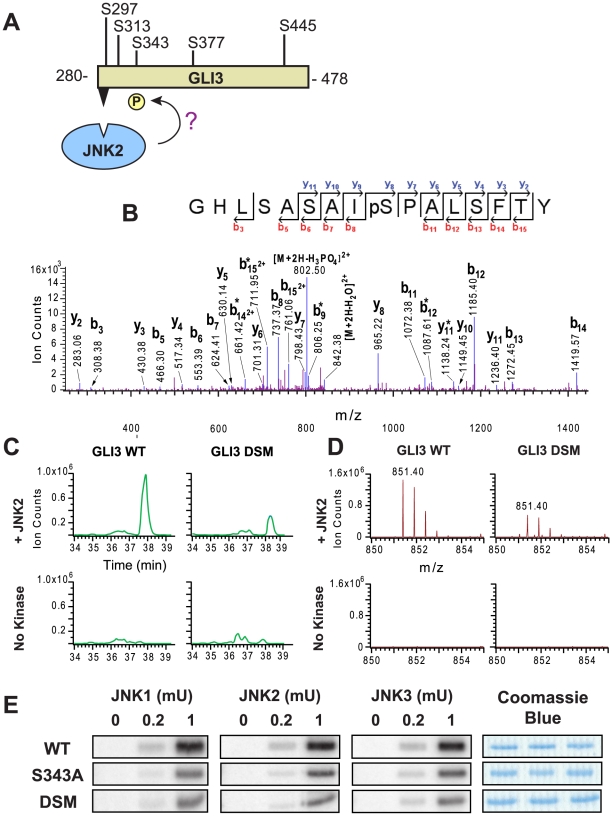
D-site-directed phosphorylation of Gli3 Ser343. (A) There are five putative MAPK target sites in the portion of Gli3 protein found to be phosphorylated in this work. To determine which sites were phosphorylated, Gli3 was incubated with active JNK2. (B), (C) & (D). Mass spectrometry analysis of an identified phosphorylated peptide (m/z 851.40, GHLSASAIS(phospho)PALSFTY). Four samples were analyzed: WT Gli3 with active kinase, DSM with active kinase, WT with no kinase, and DSM with no kinase. (B) MS/MS spectra of the identified peptide in the GST-GLI3_280–478_ WT plus active kinase sample. bi* = [bi−H3PO4]; yi* = [yi−H3PO4]. (C) Extracted ion chromatograms (XIC) of the parent ion from the four samples during LC MS runs. (D) MS of parent ion. (E) Results of an in vitro kinase assay assessing the phosphorylation of the WT, S343A, and DSM fragments of GLI3_280–478_ by activated JNK1, JNK2 and JNK3. Image is representative of three separate trials. The Coomassie Blue stained panels demonstrate equal loading of substrates. Substrate concentration: 1 µM. Enzyme activity: 0, 0.2, or 1 mU.

Within the Gli3_280–478_ fragment, there are five possible MAPK target phosphosites (S/T-P; Fig. 8A). To identify site(s) phosphorylated by JNKs in a D-site-promoted manner, we used tandem mass spectrometry to compare the phosphorylation of four different samples, consisting of the wild-type and D-site mutant versions of Gli3_280–478_, incubated either with or without active JNK2 and ATP. Following real or mock phosphorylation reactions, the products were separated by SDS-PAGE, digested with chymotrypsin, and analyzed by LC MS/MS. MS/MS analysis of the wild-type sample in the presence of kinase identified a phosphopeptide (MH_2_^2+^ 851.40) with a sequence of GHLSASAI(pS)PALSFTY (Fig. 8B). As shown in the MS/MS spectrum (Fig. 8B), the fragment ion (MH_2_^2+^ 802.50) derived from the parent ion with a loss of H_3_PO_4_ was observed, indicating that this peptide is phosphorylated. The detection of a series of y ions (i.e., y_2_∼y_11_) and b ions (i.e., b_3_, b_5_–b_8_, b_11_–b_15_) identified the peptide sequence unambiguously. In order to compare the identified phosphorylation in the four different samples, extracted ion chromatograms of the phosphorylated peptide (MH_2_^2+^ 851.40) were obtained and compared as shown in Fig. 8C. A prominent peak, eluting at around 38 minutes, is present in the phosphorylated wild-type sample, diminished in phosphorylated D-site-mutant sample, and absent in the unphosphorylated control samples. The mass spectra of this peak at the given elution time (Fig. 8D) further demonstrated that the identified phosphopeptide (MH_2_^2+^ 851.40) was only present in the samples with kinase and absent in the no kinase controls. Taken together, these results suggested that phosphorylation of Ser343 in the GST-Gli3_280–478_ protein was mediated by the D-site.

To validate the mass spectrometry results, we used site-directed mutagenesis to change Ser343 to alanine and repeated the *in vitro* kinase assay with active JNK1, JNK2, and JNK3. The result of this assay (Fig. 8E) showed a reduction in phosphorylation of the GST-Gli3_280–478_ S343A protein to the low levels seen with the D-site mutant protein, confirming the mass spectrometry results. However, there was still detectable JNK-mediated phosphorylation in the S343A mutant. This could indicate that there is at least one additional authentic target residue in this region (and that the phosphorylation of this residue(s) is not strongly dependent on the integrity of the D-site). Alternatively, it is possible that removal of the D-site leads to non-authentic phosphorylation of cryptic sites, as has been observed with the JNK substrate c-Jun [18]. These possibilities are currently under investigation.

ERK2-mediated phosphorylation of the S343A mutant of Gli3_280–478_ was also reduced compared to the wild type; however, it was not reduced all the way down to the level seen with the D-site mutant (data not shown). This suggests that Erk2 phosphorylates Ser343 and at least one additional residue in a D-site dependent manner.

To summarize, we have identified Gli3 as a novel substrate of JNK and ERK. The predicted D-site mediates binding of Gli3_280–478_ to MAPKs, and also promotes the JNK-mediated phosphorylation of Ser343, as well as the ERK-mediated phosphorylation of both S343 and an additional target site(s).

### Gli1 is also a MAPK substrate

**Figure 9:**
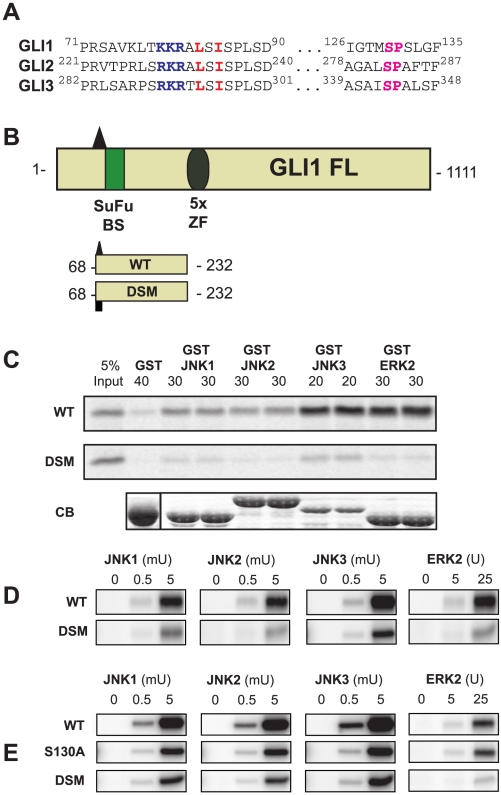
Gli1 is a novel MAP kinase substrate with a functional D-site. (A) Sequence alignment of Gli1 and 2 with Gli3 in the regions around the validated D-site (282–301 in Gli3) and target phosphosite (Ser 343 in Gli3). (B) Diagram of full length Gli1, shown with its Suppressor-of-Fused binding site (SuFu BS, green rectangle), and its 5 Zinc Finger (ZF) DNA-binding domains (gray oval). The position (triangle) and sequence of the D-finder-predicted D-site is also shown. Below, the Gli1_68–232_ wild-type (WT) and D-site mutant (DSM) fragments used for binding and kinase assays are shown. (C) The Gli1_68–232_ wild-type and D-site mutant proteins were tested for binding to GST, GST-JNK1, GST-JNK2, GST-JNK3, and GST-ERK2 (40, 30, 30, 20 and 30 µg respectively). The upper two panels show the bound Gli1 derivatives, with 5% if the total input shown in lane 1; the lower panel shows Coomassie Blue (CB) staining of the sedimented GST-fusion proteins. Other details as in Fig. 4C. (D) *In vitro* kinase assays assessing the phosphorylation of the WT and DSM fragments of Gli1 by active JNK1, JNK2, JNK3 and ERK2. Three separate concentrations of substrate (0.1, 0.3 and 0.5 µM) were incubated with the indicated units of active enzyme. Image is representative of three independent experiments. Other details as in Fig. 4E. (E) As in D, but with the addition of the S130A mutant of Gli1.

Gli3 is one of three mammalian homologues of the *Drosophila* transcription factor Ci [83]. Gli1, the founding member, was initially identified as being highly amplified in gliomas, and Gli2 and Gli3 were subsequently cloned by hybridization [83]. A D-site in Gli2 was identified by D-finder as having a high-ranking score (3E-22, rank 234 of 403), while the D-site in Gli1 received a score of 2E-24, not far below our cutoff of 1E-23. The three Gli proteins contain regions/domains of homology with each other, separated by stretches of divergence [84]. Notably, the putative D-sites in all three proteins reside in a conserved region (Fig. 9A), and share extensive sequence similarity, particularly among the key basic and hydrophobic residues.

Although all three Gli proteins are implicated in stem cell biology, development and disease pathogenesis [85], [86], evidence suggestive of crosstalk with MAPK signaling is especially enticing for Gli1 [76], [77], [78], [79], [80], [81], [82], particularly in relation to a role in tumorigenesis and cancer progression [87], [88]. Thus, to study the role of the putative Gli1 D-site in MAPK-mediated transactions, we produced wild-type and D-site-mutant fragments corresponding to the fragments we generated for Gli3. These polypeptides run from the putative D-site to just before the start of the zinc finger domain repeats (residues 68–232, Fig. 9B). As shown in Fig. 9C, wild-type Gli1_68–232_ bound to all MAPKs tested (JNK1-3 and ERK2), and bound particularly well to JNK3 and ERK2. In addition, the D-site-mutant of Gli1_68–232_ exhibited substantially impaired binding (Fig. 9C) to MAPKs. When incubated with active JNK1, JNK2, JNK3 or ERK2, wild-type GST-Gli1_68–232_ was phosphorylated with high efficiency, whereas the D-site mutant of the Gli1 fragment was phosphorylated to a much lower extent (Fig. 9D). These data indicate that the conserved D-site in Gli1 promotes binding and phosphorylation by JNKs and ERK2.

As detailed in the previous section, our mass spectrometry and mutagenesis analysis revealed that Ser343 of Gli3 was phosphorylated by JNKs. This region of Gli3 is conserved in Gli1 (and Gli2), and Ser343 in Gli3 aligns with Ser130 in Gli1 (see Fig. 9A and also Fig. 4 in Text S1). Thus it seemed reasonable to hypothesize that Ser130 in Gli1 might be a target for MAPK-mediated phosphorylation. To test this possibility, an S130A mutant of Gli1_68–232_ was constructed, purified, and incubated with active MAPK enzymes and ATP in kinase assays. Fig. 9E shows that for JNK1, JNK2 and JNK3-mediated phosphorylation, removal of Ser130 in Gli1 resulted in a reduction of phosphate incorporation down to the low levels seen in the D-site mutant. This result indicates that Ser130 is the major target phosphosite for D-site-directed JNK phosphorylation of Gli1_68–232_.

Similar to the results seen with ERK2 phosphorylation of Gli3 S243A, there was considerable ERK-mediated phosphorylation of GST-Gli1_68–232_ even when Ser130 was mutated to alanine. This contrasts with the substantial reduction in ERK-mediated phosphorylation seen with the D-site mutant of Gli1. Thus, the D-site in Gli1 must primarily direct ERK2 to target residue(s) other than Ser130. Indeed, there are six other SP sites in Gli1_68–232_ that could be ERK target phosphosites.

To summarize, we have identified Gli1 as a novel substrate of JNK and ERK. JNK phosphorylation of Ser130 in Gli1 is mediated by the predicted and experimentally verified D-site; however, this same D-site directs ERK2 to phosphorylate a distinct target site(s) in Gli1.

### Biochemical validation of additional selected candidates

Other candidates that gave a positive binding signal on the peptide array were also tested in binding assays. The histone-lysine N-methyltransferase Mixed Lineage Leukemia 4 (MLL4), a 2715-residue transcriptional regulator that is a potential oncogene [89], had a predicted D-site ranked number 15 overall by D-finder, and displayed 161% normalized binding on the peptide array. Two C-terminal fragments of this protein, one with, and one without, the putative D-site were constructed (see Fig. 5A in Text S1). In binding assays (see Fig. 5B in Text S1) the wild-type fragment bound to JNK1 and JNK2, while the fragment lacking the D-site did not. As the region near the D-site was devoid of potential MAPK phosphosites, further analysis of MLL4 was not pursued.

A putative D-site in Nei endonuclease VIII-like 1 (Neil1), a 390 amino acid protein involved in DNA repair [90], was ranked number 8 overall by D-finder, and bound at 198% of the level of the MKK7-D2 positive control on the peptide array. As shown in Fig. 5C and D in Text S1, full-length Neil1 protein bound to GST-JNK1, but not to JNK2, while the corresponding D-site mutant bound to neither JNK1 nor JNK2. Neil1, however, was not an efficient substrate for JNK-mediated phosphorylation (data not shown).

There were other cases where the predicted D-site had no apparent effect on the larger protein's ability to bind to JNK. In several cases the larger protein did not bind to JNK (or exhibited barely detectable binding) even though the predicted D-site peptide did bind to JNK in the peptide array assay. These cases included ARG1 (see Figs. 5E, F in Text S1), PIK3R1, SH3RF1/POSH, FARP2, PKD1, and WNK1 (data not shown). A possible explanation for this class of results is that the predicted D-site was either buried or locked into position in the native structure and thus not available for MAPK binding. In other cases, the full-length protein bound to JNK, but this binding was not strongly affected by mutation of the predicted D-site (e.g. PIF1, IRGQ, H6PD, PLEKHA6, LAG3, DLEC1, CNKSR, NR4A, MIB2, data not shown). In total, 21 proteins were given full biochemical workup to the point of being tested in binding assays. Six of these 21 (hnRNP-K, PPM1J, Gli3, Gli1, MLL4 and Neil1) exhibited D-site dependent binding, and for the first 4 of these 6 we found strong evidence of D-site-dependent phosphorylation. For 15 of the 21 biochemically-tested proteins (∼70%), in contrast, we were unable to find evidence that the predicted D-site was strongly functional for binding in the context of the native polypeptide. In at least some of these cases, it is possible that the predicted D-site is redundant with other docking sites in the same polypeptide, or that the predicted D-site might be more active in a kinase assay than in a binding assay.

## Discussion

Currently, substantial progress is being made in the computational and functional genomic investigation of the phosphoproteome [11], [91], [92], [93]. However, considering that in humans about 500 protein kinases must phosphorylate over 6000 proteins on multiple sites, it is clear that much still remains to be learned. Here we have developed D-finder, a computational tool that uses a hybrid pattern matching/hidden Markov model algorithm to search protein sequences for kinase-substrate docking sites. The first version of D-finder focuses on docking sites for the JNK family of mitogen-activated protein kinases. Using this tool, we identified previously undiscovered D-sites in the known JNK substrates hnRNP-K and PPM1J. We also identified functional D-sites in the DNA repair protein Neil1, and near the C-terminus of the histone-lysine N-methyltransferase MLL4. Finally, we identified the hedgehog pathway transcription factors Gli1 and Gli3 as novel, D-site-dependent JNK and ERK substrates. The latter observations, if verified *in vivo*, could be important for understanding crosstalk and integration between MAPK and hedgehog pathways in stem cell biology, development and cancer.

### D-finder

Accurate computational prediction of protein kinase target phosphorylation sites (phosphosites) is limited by the short length and consequent degeneracy of these sites. Nevertheless, predictions based on phosphosite specificity have been successful for some kinases (e.g. [9], [10], [11]). For other kinases, however, such as MAP kinases, this approach has not been as fruitful. Here we have attempted to leverage the observation that several important families of kinases, including MAPKs, tether or dock themselves to their substrates prior to phosphorylating them [13], [14], [15], [16], [17]. These docking interactions are mediated by docking motifs on substrates that, while still relatively short, potentially contain more information then phosphosites. We employed a profile hidden Markov model –a data-driven machine learning approach– to infer as much information as possible from a training set of literature-verified docking sites. Although this model (D-learner) performed well in multiple validation tests, when run on the human genome, it made many high-ranking predictions that were inconsistent with expert knowledge of D-site structure/function that had been gleaned from site-directed mutagenesis studies. To ameliorate this problem, D-matcher, a simple pattern-matching scheme, was added as a pre-screen to find peptides suitable to pass to D-learner. This addition was computationally trivial, yet quite effective, judging on the ability of the resulting hybrid procedure, D-finder, to predict binding peptides (see below). More elaborate hybrid HMM approaches have been applied to other sequence analysis tasks, e.g. hybrid HMM/neural networks [94], [95].

When run on the translated human transcriptome, D-finder found 403 above-threshold D-sites, about 8-fold the number that would be predicted by chance. Based on the peptide array assay (Fig. 6), D-finder was remarkably adept at identifying *bona fide* docking site peptides: about 3/4 of the D-finder-predicted peptides we tested bound to JNK1 at a level that exceeded the binding of the known D-site MKK7-D2. Of course, just because a given sequence can bind to JNK as a peptide, this does not necessarily imply that it will bind in the context of the native protein. Indeed, when worked up to the level of binding assays, ∼30% of candidates tested were found to bind to one or more of the JNK proteins in a D-site-dependent manner. It should be noted that D-site-dependent binding is a relatively stringent assay for D-site functionality; D-sites with very low affinity in binding assays can still effectively direct MAPKs to phosphorylate particular target residues (our unpublished observations). In addition, two or more docking sites can function redundantly with each other; thus the functionality of a particular docking site may be missed. Nevertheless, taking the numbers at face value, as 3 of 4 predictions passed the peptide array test, and roughly 30% of these passed full biochemical workup, it can be estimated that about 1 in 5 of D-finder's predictions are true positives. This may well be an underestimate of the true positive rate for reasons given above.

In future modifications, D-finder could be improved in several ways. First and most obvious, new members could be added to the training set, including the novel D-sites identified in this work. Second, the D-matcher front end could be improved by the addition of further expert knowledge gleaned from additional mutagenesis studies. Third, the possibility of using additional information, such as predicted surface accessibility, intrinsic disorder and evolutionary conservation, could be explored [96], [97]. Finally, the algorithm could be modified to search for docking sites that are near to a putative phosphosite or to a cluster of phosphosites [98]. In addition, ERK and p38-targeted versions of D-finder could be created. MAPK docking sites are found in diverse members of the plant and animal kingdom, and have proven to be structurally and functionally conserved from yeast to humans [99]; thus D-finder could be profitably run on additional genomes.

### A D-site in hnRNP-K

D-finder identified a previously unknown D-site in heterogeneous nuclear ribonucleoprotein K (hnRNP-K), a transcriptional and translational regulator and known JNK/ERK substrate that is dysregulated in both colon cancer and leukemia [58], [59], [60], [61], [62], [63]. We showed that this D-site mediated both JNK binding and JNK-mediated phosphorylation of Ser384 (Fig. 4). Interestingly, this newly identified D-site lies within the K-protein-interactive (KI) domain, a region known to mediate protein-protein interactions between hnRNP-K and Src family kinases, PKCdelta, WASP, transcription factors, and other partners [58], [64], [65], [66]. Indeed, the D-site overlaps with SH3-binding site P3 (RARNLPLPPPPPPRGG), known to interact with c-Src and Vav [100], and contains Ser302, a target site of PKCdelta [65]. These considerations suggest that: (1) JNK may compete with other partners for binding to hnRNP-K; (2) hnRNP-K may serve as a scaffold to facilitate JNK-mediated phosphorylation of other hnRNP-K binding partners; (3) PKC-mediated phosphorylation of Ser302 may regulate JNK docking. ERK was also found to utilize the newly identified docking site, although mutation of the D-site did not strongly affect ERK-mediated phosphorylation of Ser384.

### Gli1 and Gli3 are MAPK substrates

Gli3, a transcription factor in the hedgehog pathway that is mutated in human developmental disorders [75], contained the top novel prediction made by D-finder. Verifying this prediction, we found that this D-site (located in residues 281–300 of Gli3) mediated binding to JNK1-3 and ERK2. In addition, we showed that the 281–300 D-site promoted JNK- and ERK-mediated phosphorylation of target site(s) within Gli3 residues 280–478. Finally, using mass spectrometry, we identified Ser343 as a prominent JNK phosphosite, and showed that JNK-mediated phosphorylation of this site was dependent on the integrity of the novel D-site.

D-finder also pinpointed highly similar D-sites in the paralogous transcription factors Gli1 and Gli2, proteins that (like Gli3) are important in regulators of stem cells and development, and which are dysregulated in several types of cancer [83], [85], [86]. Examining Gli1, we found that the homologous D-site (located within residues 72–91 of Gli1) mediated the binding of JNK1-3 and ERK2 to Gli1. Further, we showed that this D-site promoted JNK- and ERK-mediated phosphorylation of target site(s) within Gli1 residues 68–232, and that Ser130 (homologous to Ser343 in Gli3) was a D-site-dependent JNK phosphosite.

### Evidence for MAPK-Gli connection

There has been surprisingly little evidence for integration between the hedgehog and MAP kinase pathways, two of the major signaling pathways controlling early development [101]. Recently, however, several papers have provided evidence for such a connection, particularly in cancer (reviewed in [87], [88]). Typically, these studies have employed both MAPK pathway activation (using ligands such as epidermal growth factor or fibroblast growth factor, or using constitutively active Ras or MEK) and pharmacological inhibition to document effects on hedgehog pathway readouts such as Gli-dependent transcription, cell differentiation and proliferation [76], [77], [78], [79], [81], [82], [102], [103]. For example, Kessaris et al. found that hedgehog-stimulated differentiation of oligodendrocite progenitors required ERK activation [102]. Using a tissue culture model of basal cell carcinoma, Schnidar et al. [78] found that activation of the MEK1/2→ERK1/2 pathway synergized with Gli1 to transform cells. Other cancer types where MAPK/Gli crosstalk has been implicated in pathogenesis include medulloblastoma [103], gastric cancer [79], melanoma [82], and pancreatic cancer [81]. Indeed, Hanahan and colleagues have recently proposed that in pancreatic cancer, non-canonical RAS-mediated activation of Gli proteins is the primary mechanism of tumorigenesis [80].

Most of the above studies provide evidence for ERK involvement in Gli regulation, but do not exclude the possibility of JNK involvement as well. Positive evidence for crosstalk/integration between Gli transcription factors and the JNK MAP kinases is less extensive at present. Fogarty et al. [103] showed that fibroblast growth factor blocked sonic hedgehog signaling in neural precursor cells. This ability of FGF to inhibit the hedgehog pathway was apparently mediated by both JNK and ERK, with JNK predominating.

Intriguingly, two groups have narrowed down the region of Gli1 that responds to MAPK signaling. Riobo, et al. [76] found that Gli1 transcriptional activity was enhanced by activation of ERK, and that the first 130 residues of the 1100+-residue Gli1 protein were required for sensing the ERK pathway. Likewise, Seto et al. [79], using a similar assay, mapped the ERK-responsive regions to the first 116 residues of Gli1. Hence, two independent studies have narrowed down the ERK-responsive region of Gli1 to a small portion of the protein that contains the D-site identified in this work. This region appears to be a ‘hotspot” for the regulation of Gli activity [84], [104], [105].

To summarize, there is considerable compelling recent evidence for MAPK-mediated regulation of Gli transcription factors, and for the importance of this crosstalk in stem cell development and cancer. Our study, however, is the first to provide direct evidence that ERK or JNK either bind to or phosphorylate Gli transcription factors.

### Specificity of docking sites and docking-directed phosphorylation

A final set of observations that emerge from the present study concerns the specificity of docking site action. It has been established that different docking sites in the same substrate can direct MAPKs to distinct target phosphosites. For example, an FXFP-class docking site in Elk-1 directs ERK to phosphorylate Ser383, whereas the D-site in Elk-1 directs ERK to phosphorylate other residues [106]. Similarly, in the paralogous transcription factor Net, ERK and JNK, via different D-sites, bind to and phosphorylate separate domains of the protein [54]. Here, we found evidence of a different phenomenon: the *same* D-site can direct different MAPKs to distinct phosphosites on the same substrate. In Gli3, Ser343 received the bulk of JNK-mediated phosphorylation directed by the 281–300 D-site. In contrast, ERK phosphorylation by the same D-site was directed both to Ser343 and to other residue(s). Even more strikingly, in Gli1 Ser130 received the bulk of JNK-mediated phosphorylation directed by the 72–91 D-site, whereas this phosphosite was phosphorylated hardly at all by ERK; instead the same D-site directed ERK to phosphorylate completely different residue(s).

In conclusion, using D-finder, a tool developed to search genome databases for JNK-docking sites, we have discovered new MAPK docking sites, binding partners, and substrates, including the hedgehog-pathway transcription factors Gli1 and Gli3.

## Methods

### D-learner hidden Markov model

A profile HMM architecture, composed of linked main, insert, and delete states was implemented in the programming language Java to perform the computational analysis and prediction. An HMM model of length 19 was used to match the average length of the available docking site sequences. The initial state transition and emission probabilities were uniformly set across the model prior to training. Using the available, experimentally determined docking site sequences, the HMM was trained using Viterbi learning [34] by running each sequence through the model and updating the transition and emission probabilities accordingly. This procedure was repeated 300 times allowing convergence of the sum of the Viterbi probabilities to a constant value.

The probabilistic score produced by D-finder is technically called the Viterbi score. This score is generated by calculating the log-likelihood of the D-matcher-approved string (as a complete Viterbi path in the HMM) based on the trained model. The greater the generated score (i.e., the closer to 1), the closer the likelihood is to the optimal Viterbi path. In other words, the Viterbi score is the probability P^V^ of the most likely HMM path for the given sequence. The most likely path is a sequence of state transitions and state emissions. Each transition and each emission has a probability. These probabilities get multiplied with each other along the most likely path; as a result, Viterbi scores are typically very small numbers.

### D-matcher algorithm

The D-matcher algorithm was predicated on the following three pieces of expert knowledge, specifically drawn from general trends in experimental data on the JNK-MKK4 interaction ([32], DTH unpublished data): (1) a φ-X-φ submotif, in which the middle residue is not itself strongly hydrophobic, is optimal for high-affinity JNK binding; (2) binding affinity is proportional to the number of basic residues in the basic submotif, and gaps in between the basic residues result in decreased binding affinity (3); there is a limit to the allowable distance between these two submotifs. The first version of D-matcher first identified all hydrophobic-X-hydrophobic regions (with V, I, L, M defined as hydrophobics, and X not allowed to be a hydrophobic). Substrings 12 amino acids long preceding each ϕ-X-ϕ were then pulled out for further analysis. With each substring, a local-to-global alignment [107] was performed using a 3 basic residue motif as the local sequence; this was used to give a numerical score.

D-matcher consistently gave higher scores to the wild type MKK4 D-site than to point mutants that have been shown experimentally to reduce JNK binding affinities ([32], DTH unpublished data). Also, when tested on other known JNK-binding proteins, D-matcher correctly ranked the known D-site higher compared to other potential D-sites (data not shown).

The simplified D-matcher incorporated into D-finder was designed to be a minimal prescreen that simply checks for a basic residue followed after a spacer of 1–3 residues by a hydrophobic-X-hydrophobic (as defined above).

### D-finder algorithm

D-finder is a hybrid of D-matcher and D-learner. Specifically, a modified D-matcher is used to select suitable strings to pass to D-learner, which then assigns a standard HMM Viterbi probability score. Each full-length sequence was assigned a score equal to the score of its highest probability D-matcher-passing string. The sequences were then ranked yielding a sorted list of predicted D-sites (see Table 1 in Table S1). D-finder is written in Java; the code is downloadable from http://dfinder.sourceforge.net as a .zip file that contains the Java files along with the original training set file, a sample testing file, and a README file.

### Transcriptome screening and human genes

The translated human transcriptome was obtained from the UCSC Genome Browser (hg19; 33,730 protein sequences). The human MAPK genes used in this study were JNK1α1 (MAPK8, NCBI Accession Number NM_002750), JNK2α2 (MAPK9, NM_002752), JNK3α1 (MAPK10, NM_002753) and ERK2 (MAPK1, NM_002754). Accession numbers for the MAPK substrates examined in this work are given in Fig. 3 and Table S1.

### Peptide array

Custom synthesis of the peptide arrays used in this study was performed by JPT (Berlin, Germany), as described elsewhere [70]. The 17-mer peptides (see Table 3 in Table S1) were chemically linked to a nitrocellulose membrane via the C-terminus. Two separate array designs were synthesized twice each. Design 1 had four control spots (two positive, two negative) and 42 predicted D-site peptides synthesized in duplicate (total spots/array = 96). Design 2 had the same four controls, 18 training set D-site peptides, and 30 predicted D-site peptides synthesized in duplicate (total spots/array = 104). The membrane was probed with [^35^S]-methionine labeled JNK1 as described elsewhere [32].

### Biochemical workup

An outline of our strategy to efficiently test selected candidates is as follows: cDNA clones of candidates were obtained from the mammalian gene collection [108] where available (in some cases only fragments were available). Open reading frames were subcloned downstream of a bacteriophage RNA polymerase promoter, and the encoded protein was produced in radiopure form by coupled *in vitro* transcription/translation, as described elsewhere [109]. The *in vitro*-translated candidate protein was then used in a binding assay (*a.k.a.* a GST pull-down assay) with various GST-MAPKs. If a candidate exhibited MAPK binding, the predicted D-site was mutated or deleted and the mutant protein was retested. Selected candidates that exhibited D-site-dependent binding were then subcloned into bacterial expression vectors as GST-fusions. This step often involved considerable optimization to find a suitable fragment that was expressed as soluble protein at a reasonable yield. Purified GST-fusion proteins were then used as substrates in protein kinase assays with purified active MAPK enzymes.

### Plasmids for in vitro transcription and translation

cDNA clones from mammalian gene collection [108] were obtained from Open Biosystems (Huntsville, AL) or OriGene (Rockville, MD). The Gli3 plasmid was a gift of Dr. Bert Vogelstein, Johns Hopkins University. Regions of interest were amplified by PCR using *Pfu* Ultra DNA polymerase (Stratagene). PCR products were purified (Qiagen) and digested with restriction enzymes designed into the primers, run on 1% agarose gels, and excised for gel extraction (Qiagen). Digested, gel purified products were inserted into pGEM-4ZStop, a variant of pGEM4Z that contains multiple stop codons downstream of the cloning site (a gift from A. Jane Bardwell of this laboratory). Plasmid sequences were verified by DNA sequencing (Cogenics). To create D-site mutant (DSM) constructs, basic and hydrophobic residues in the predicted D-site were substituted with alanine residues using appropriate primers and the Quickchange site-directed mutagenesis kit (Stratagene). Mutations were verified by DNA sequencing.

### Plasmids for the production of GST fusion proteins and cell culture

Open reading frames of interest were subcloned into pcDNA 3.1 (+) and pGEX-LB (a derivative of PGEX-4T-1) as described [99]. New primers were designed for amplification of smaller fragments, where appropriate.

### *In vitro* transcription and translation

Proteins labeled with [^35^S]-methionine were produced by coupled transcription and translation reactions (SP6, Promega). Translation products were partially purified by ammonium sulfate precipitation [109] and resuspended in Binding Buffer (20mM Tris-HCl (pH 7.5), 125mM KOAc, 0.5mM EDTA, 1mM DTT, 0.1% (v/v) Tween20, 12.5% (v/v) Glycerol). Comparable translation products were normalized for GST pull down assays by SDS-PAGE and quantification using a Typhoon PhosphorImager (Amersham Biosciences).

### GST pull down assays

Comparable amounts of [^35^S]-methionine labeled proteins (i.e., wild-type vs. D-site mutant) were pre-cleared against BSA-blocked glutathione Sepharose beads, then incubated with GST-MAPK fusion proteins or GST alone at 30°C for 15 min followed by an additional 30 min at room temperature with gentle rocking. Complexes were then sedimented, washed extensively with binding buffer, and heated in reducing SDS sample buffer. Samples were separated by SDS-PAGE, fixed in 40% Methanol/12% Acetic Acid, Coomassie Blue stained (using Gelcode Blue, Pierce), dried, visualized and quantified using a PhosphorImager. To generate values for percent binding, bands from experimental lanes were normalized to the 5% input lane.

### Purification of recombinant GST proteins

Expression of recombinant GST fusion proteins was induced in *Escherichia coli* BL21 cells (Stratagene) at 30°C for 2 h by addition of 1-thio-β-D-galactopyranoside [IPTG, 0.6 mM final]. Cell pellets were resuspended in lysis buffer (1× PBS, 1mM EDTA, 5mM DTT, 0.1% Triton, 1mM PMSF, 15% Glycerol), and the resulting extract was sonicated, clarified with 20% Triton X-100, and centrifuged at 12,000×g for 10 min to remove cell debris and nucleic acids. GST fusion proteins contained within the supernatants were purified by affinity chromatography using glutathione-Sepharose (Amersham Biosciences), eluted from beads using 10mM reduced glutathione, and dialyzed overnight against lysis buffer. Eluted proteins were quantified against BSA standards.

### Protein kinase assays

Kinase reactions (20 µl) contained 1× MAP Kinase Buffer (50 mM Tris-HCl (pH 7.5), 10 mM MgCl_2_, 1 mM EGTA, and 2 mM DTT), 50 µM ATP, 1 µCi of [γ-^32^P]-ATP, enzyme, and substrate. Enzymes were: JNK1α1, active; JNK2α2, active (Upstate); JNK3/SAPK1b, active (all from Upstate Biochemicals/Millipore); ERK2, active (New England Biosciences). Substrates were: GST-hnRNP-K w/D, 1 µM (896 ng/20 µl); GST-hnRNP-K w/oD, 1 µM (818 ng/20 µl); GST-PPM1J WT and DSM, 0.5 µM (750 ng/20 µl); GST-Gli3_280–478_ WT and DSM, 0.5 µM (465 ng/20 µl); GST-Gli1_68–232_ WT and DSM, 1 µM (856 ng/20 µl). Reactions were incubated at 30°C for 30 min, then stopped with SDS sample buffer, separated by SDS-PAGE, fixed in 40% Methanol/12% Acetic Acid, Gelcode Blue stained, dried, and visualized using a PhosphorImager. Unit definitions for enzymes were as supplied by the manufacturer. Note that the unit definition for ERK2 and the JNK proteins are different. For ERK2, 10 units is about 1 ng of enzyme, corresponding to a concentration of 1.2 nM in a 20 µl reaction. For the JNK proteins, 1 ng of enzyme corresponds to about 0.5 milliunits (mU).

### Tissue culture and transfection

Cos-1 cells were cultured using Dulbecco's modified Eagle's medium enriched with 10% heat-inactivated fetal bovine serum (Invitrogen), penicillin, streptomycin, and sodium bicarbonate. The cells were seeded at a density of 3×10^5^ cells per well in a 6-well dish in antibiotic free media. The culture was maintained in a humidified environment at 37°C and 5% CO_2_. Transient transfection was performed with Lipofectamine (Invitrogen) following the manufacturer's recommended procedures.

### Immunoprecipitation kinase assays

Cos-1 cells were transfected with 1 µg of plasmid DNA encoding either V5-tagged wild-type (WT) PPM1J, docking site mutated (DSM) PPM1J, or empty vector. After 16 h, the cells were harvested and lysed in 200 µl HEPES Lysis Buffer (HLB, 20 mM Hepes (ph 7.4), 137 mM NaCl, 2 mM EDTA, 10% glycerol (v/v), 1% Triton X-100 (v/v), 25 mM β-glycerophosphate, 1 mM Sodium Vandate, 1∶100 protease inhibitor cocktail (Sigma)) and centrifuged at 14,000×g for 15 min at 4°C. Forty µl of each supernatant was removed for immunoblotting. The PPM1J WT and D-site mutant supernatants were cleared with 20 µl of a 50% slurry of Protein G Plus/Protein A-agarose beads for 30 min at 4°C. The cleared lysates were incubated for 30 min at 4°C with 2 µl of anti-V5 antibody (Invitrogen). Twenty µl of beads (50% slurry) were added and incubated overnight at 4°C. Complexes were sedimented and then washed twice with HLB plus 0.1% Triton X-100 and once with MAP Kinase buffer. The immunoprecipitated complexes were then used in a kinase assay with 1 mU of active JNK1α1.

### Liquid chromatography-tandem mass spectrometry (LC MS/MS)

Phosphorylation reactions were as described above except that ATP was raised to 200 µM and the radioactive ATP tracer was omitted. Following real or mock phosphorylation reactions, the products were separated by SDS-PAGE, and bands corresponding to the mass of the Gli3 fragments were excised from the gel and digested with chymotrypsin. The resulting peptide digests were extracted and analyzed by LC MS/MS as described [110]. Briefly, the LC analysis was performed using a capillary column (100 µm ID×150 mm long) packed with C18 resins (GL Sciences) and the peptides were eluted using a linear gradient of 2–35% B in 35 min; (solvent A: 100% H2O/0.1% formic acid; solvent B: 100% acetonitrile/0.1% formic acid). A cycle of one full FT scan mass spectrum (350–1800 m/z, resolution of 60,000 at m/z 400) was followed by ten data-dependent MS/MS acquired in the linear ion trap with normalized collision energy (setting of 35%). Target ions selected for MS/MS were dynamically excluded for 30 s.

Protein identification and characterization was carried out by database searching using Protein Prospector [110]. LC MS/MS data was extracted, and submitted to database searching using the Batch-Tag against a decoy database consisting of a normal SwissProt database including the engineered Gli3 sequences concatenated with its random version. The mass accuracy for parent ions and fragment ions were set as ±20 ppm and 0.5 Da, respectively. Phosphorylation of Serine and Threonine was selected as the variable modification. MS/MS spectra of phosphorylated peptides were inspected manually.

## Supporting Information

Table S1 List of top D-finder predictions.
(0.22 MB XLS)

Text S1 Supplementary figures.
(0.31 MB PDF)
