# Supplementary material for: Computational Prediction and Experimental Verification of New MAP Kinase Docking Sites and Substrates Including Gli Transcription Factors
Source: PLoS Comput Biol. 2010 Aug 26;6(8):e1000908. doi: 10.1371/journal.pcbi.1000908 (PMC2928751; doi:10.1371/journal.pcbi.1000908)
Supplement: Text S1 — Supplementary figures. (0.31 MB PDF) [file pcbi.1000908.s002.pdf]

Supplemental figures for Whisenant *et al*., 2010

Computational prediction and experimental verification of new MAP kinase docking sites and substrates including Gli transcription factors

This supplemental files includes

Item #	Description	Page
Fig. 1.	Multiple sequence alignment of the D-sites in the training set.	2
Fig. 2.	Receiver operating characteristic curve for D-learner.T1	3
Fig. 3.	Examples of high-scoring D-learner.T1 predictions in absence of pre-screen by D-matcher.	4
Fig. 4.	Alignment of Gli1,2 and 3 in region around D-site and JNK phosphosite	4
Fig. 5.	Binding data for MLL4, Neil1 and ARG1	5

Supplemental figures for Whisenant *et al*., 2010

Fig. 1. Multiple sequence alignment of the D-sites in the training set.

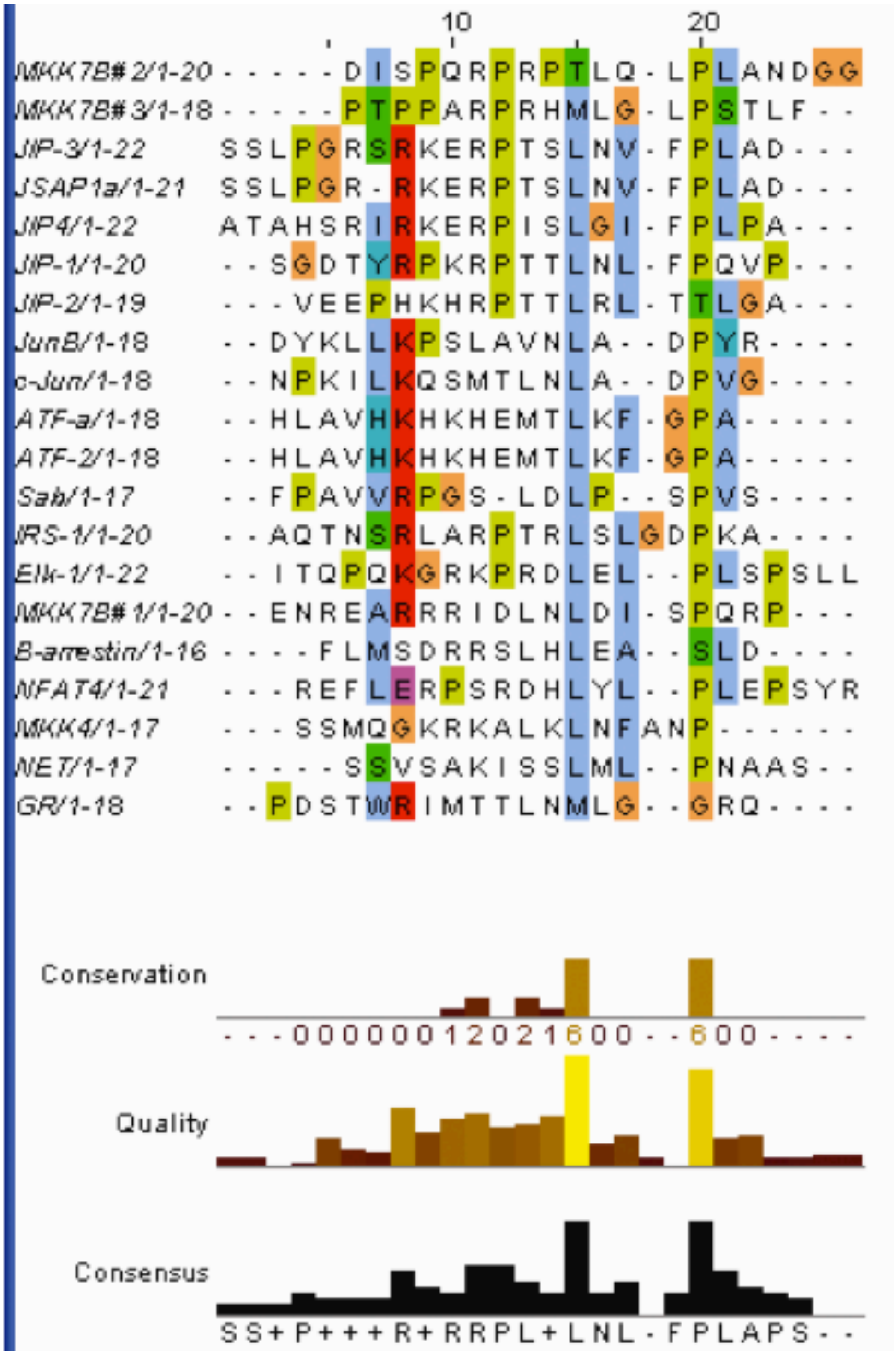

Notes: Multiple sequence alignment by ClustalW2 (www.ebi.ac.uk/Tools/clustalw2). Note that the hydrophobic-X-hydrophobic submotifs are not “correctly” aligned in all cases.

Supplemental figures for Whisenant *et al*., 2010

Fig. 2. Receiver Operating Characteristic (ROC) curve for D-learner.T1

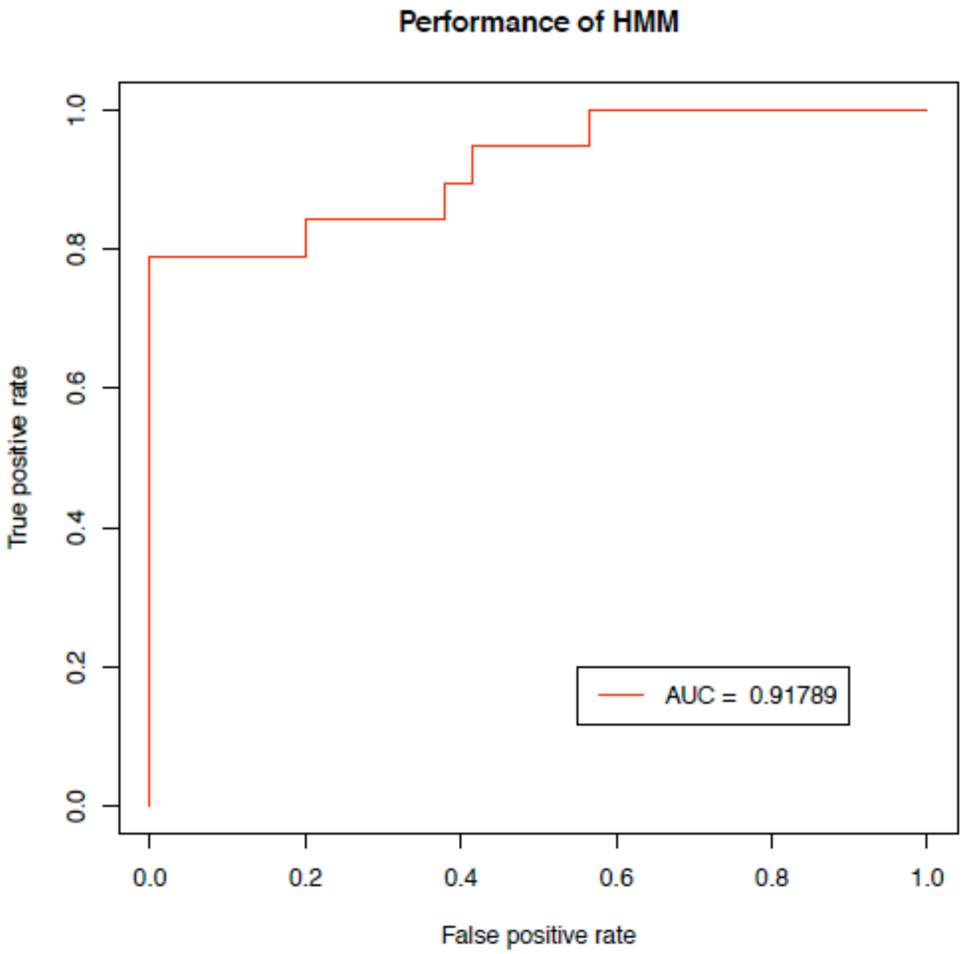

Fig. S2. Receiver Operating Characteristic (ROC) analysis, which compares how the true positive and false positive rates vary as the threshold used to discriminate predicted positives from predicted negatives is varied. To estimate the false positive rate, we used approximately 60,000 sequence windows chosen randomly from the predicted proteomes of *E. coli* and *B. subtilus*. (As bacteria do not have MAP kinases, and so any D-site-like sequences in their proteomes can be considered false positives.) For a set of true positives we used the training set members. The false positive and true positive rates were then determined as a function of the threshold Viterbi score used to separate hits from non-hits.
Conclusion: The area under the curve is about 0.92, which indicates very good performance of a classifier.

Supplemental figures for Whisenant *et al*., 2010

Figure 3.
Examples of high-scoring sequences ranked by D-learner that are filtered out by D-matcher.

Gene	Score	Sequence
PRELP	2.7E-19	FPQPDEPAEPTDLPPPLPP
MGRN1	7.7E-20	SLASKKPKRETNSDSVPPG
LTBP2	7.5E-20	REPPGRGSRPRALLEAPLK
ARHGAP25	6.7E-20	DLAVVKPTRPNSLPPNPSP

Conclusion: None of the sequences have a hydrophobic-X-hydrophobic, and PRELP doesn't even have a single basic residue. D-matcher looks for a basic residue followed shortly by a hydrophobic-X-hydrophobic, and thus would not pass these sequences.

Figure 4.

Alignment of human Gli1, Gli2 and Gli3 in the region around the D-site and the identified JNK phosphosite.

```
                D-site                                             SuFu      Pn
GLI1  71 PRSAVKLTKKRALSISPLSDASLDLQTVIRTSPSSLVAFINS--RCTSPGGSYGHLSIGTMSPSLGFPAQMN 140
GLI2 221 PRVTPRLSRKRALSISPLSDASLDLQRMIRTSPNSLVAYINNSRSSSAASGSYGHLSAGALSPAFTFPHPIN 292
GLI3 282 PRLSARPSRKRTLSISPLSDHSFDLQTMIRTSPNSLVTILNNSRSSSSASGSYGHLSASAISPALSFTYSSA 353
```

The D-site identified in this work is highlighted in blue and red, the JNK phosphosite identified in this work is are pink, and the suppressor of fused (SuFu) binding motif is in purple.

Conclusion: The D-site is conserved in all 3 Gli paralogs, as is the Gli S343 phosphosite, identified in this work as a target of JNK-mediated phosphorylation. The position of both these motifs relative to the SuFu-binding site is also conserved.

Figure 5 (next page)

(A) Diagram of MLL4 fragments tested.
(B) Binding of MLL4 fragments to GST-JNK1 and GST-JNK2. The fragment containing the D-finder-predicted D-site binds to both JNK1 and JNK2, and the fragment lacking the D-site does not.
(C) Diagram of full-length, wild-type Neil1 protein and the D-site mutant.
(D) Binding of Neil1 species to GST-JNK1 and GST-JNK2. Wild-type Neil1 binds to JNK1, whereas the D-site mutant exhibits reduced binding.
(E) Diagram of full-length Arginase 1 protein showing the D-finder-predicted D-site.
(F) Arginase 1 does not bind to JNK1 or JNK2**.**

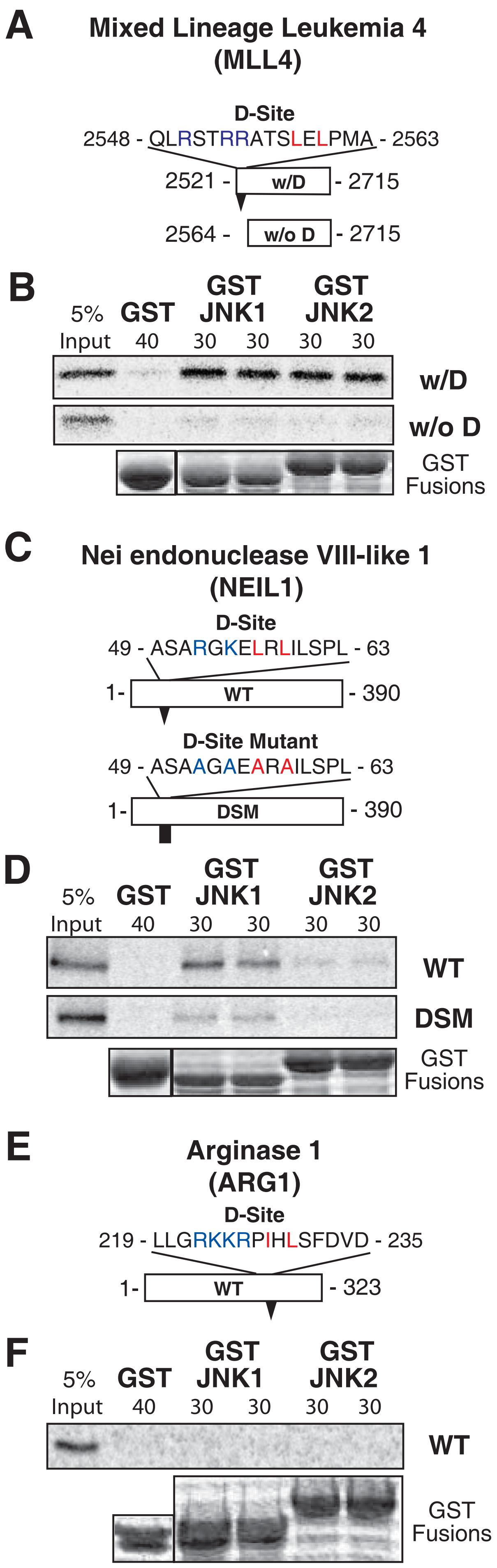
A
Mixed Lineage Leukemia 4
(MLL4)
D-Site
2548 - QLRSTRRATSLELPMA - 2563
2521 - w/D - 2715
2564 - w/o D - 2715
B
5% Input
GST 40
GST JNK1 30 30
GST JNK2 30 30
w/D
w/o D
GST Fusions
C
Nei endonuclease VIII-like 1
(NEIL1)
D-Site
49 - ASARGKELRLILSPL - 63
1- WT - 390
D-Site Mutant
49 - ASAAGAEARAILSPL - 63
1- DSM - 390
D
5% Input
GST 40
GST JNK1 30 30
GST JNK2 30 30
WT
DSM
GST Fusions
E
Arginase 1
(ARG1)
D-Site
219 - LLGRKKRPIHLSFDVD - 235
1- WT - 323
F
5% Input
GST 40
GST JNK1 30 30
GST JNK2 30 30
WT
GST Fusions
